# ANN Assisted-IoT Enabled COVID-19 Patient Monitoring

**DOI:** 10.1109/ACCESS.2021.3064826

**Published:** 2021-03-09

**Authors:** Geetanjali Rathee, Sahil Garg, Georges Kaddoum, Yulei Wu, Dushantha Nalin K. Jayakody, Atif Alamri

**Affiliations:** 1 Department of Computer Science and EngineeringJaypee University of Information Technology 29067 Waknaghat 173234 India; 2 Electrical Engineering DepartmentÉcole de Technologie SupérieureUniversité du Québec 5623 Montréal QC H3C 1K3 Canada; 3 College of Engineering, Mathematics and Physical SciencesUniversity of Exeter 3286 Exeter EX4 4QF U.K.; 4 School of Computer Science and RoboticsNational Research Tomsk Polytechnic University (TPU) 634034 Tomsk Russia; 5 Centre for Telecommunication Research, Faculty of Engineering & TechnologySri Lanka Technological Campus Padukka 11500 Sri Lanka; 6 Chair of Pervasive and Mobile Computing, College of Computer and Information SciencesKing Saud University 37850 Riyadh 11543 Saudi Arabia

**Keywords:** Artificial neural network, back propagation network, multi-perceptron layer, security in healthcare, COVID 19 patients’ identification

## Abstract

COVID-19 is an extremely dangerous disease because of its highly infectious nature. In order to provide a quick and immediate identification of infection, a proper and immediate clinical support is needed. Researchers have proposed various Machine Learning and smart IoT based schemes for categorizing the COVID-19 patients. Artificial Neural Networks (ANN) that are inspired by the biological concept of neurons are generally used in various applications including healthcare systems. The ANN scheme provides a viable solution in the decision making process for managing the healthcare information. This manuscript endeavours to illustrate the applicability and suitability of ANN by categorizing the status of COVID-19 patients’ health into infected (IN), uninfected (UI), exposed (EP) and susceptible (ST). In order to do so, Bayesian and back propagation algorithms have been used to generate the results. Further, viterbi algorithm is used to improve the accuracy of the proposed system. The proposed mechanism is validated over various accuracy and classification parameters against conventional Random Tree (RT), Fuzzy C Means (FCM) and REPTree (RPT) methods.

## I. Introduction

The onset of Coronavirus disease (COVID-19) has pushed the world into a serious jeopardizing situation and the infectious disease has already been classified by the World Health Organization (WHO) as a pandemic. The virus has been categorised as severe acute respiratory syndrome coronavirus 2 (SARS-CoV-2) due to its visual similarity to the SARS-CoV-1 [1], [2]. It is a highly contagious disease which spreads very rapidly and in order to end the pandemic, the whole world is working with all its might. Researchers are motivated to fight against COVID-19 by exploring, understanding and devising new treatments and techniques to terminate it from our current generation. In addition, COVID-19 is severely endangering the healthcare mechanisms because of its severe infectious nature. Severity infection quantification is needed to provide a proper and immediate clinical support to critical COVID-19 patients [3]. According to a WHO study, there are approximately 10.5 M confirmed cases, 10.1 M recovered cases and 151k deaths. Such cases have been detected in almost the entire world from Germany, Italy, UK and USA to India, Japan and the Korean Peninsula [4]–[6]. The prompt symptoms to detect COVID-19 patients are tiredness, eye redness, fever, cold, throat infection, respiratory problems etc. These symptoms habitually appear within 4-5 days after a person is infected.

Presentl, remote monitoring and early detection schemes to control the disease are highly desirable. Although, COVID-19 diagnostic test and IRTC tests are helping the patients to detect the virus accurately, however, these test are not much effective and prompt in stop the spreading of the disease [7]–[9]. Till now, no specific vaccine or medicines are available to cure COVID-19, though American labs have realised an antidote but it is very critical to analyze its side effects from initial use. Apart from that, there is a lack of communication and involvement between patients and doctors. In such scenarios, efficient controlling and diagnosis of virus at remote sites and homes has added a new challenge for healthcare providers.

### A. Motivation

The immense potential of cloud computing, mobile computing and IoT sensors has made it possible to design smart cloud-based health care systems [10]–[19]. Various healthcare systems have moved to IoT-based techniques to explore new benefits and services of smart mentoring and record storing of information [20], [21]. However, IoT applications generate a huge data that is further difficult to analyze and generate immediate reports of the patients. Recent smart technology systems are not able to provide real time monitoring, servicing and protection methods in hospitals, schools or crowded areas [22]. Along with numerous success stories in the biomedical and clinical diagnostic fields, Artificial Intelligence (AI) assistance can be considered as a medium to conduct quantification and remote automation of COVID-19 patients [23]–[25]. Although researchers have proposed various cloud-based, neural network and deep learning schemes by deploying the automated tomography and chest radiography. However, the advanced visualization and sensitivity provisions provide reliable CT-based screening tests as compared to traditional methods. In addition, the huge number of asymptomatic patients and the early stage detection of COVID-19 is still considered as a critical challenge because of undistinguished, small, obscure and scattered infectious regions [26], [27].

In addition, the lack of reliable data sets may further enhance the criticality of patient recognition at its early stages. AI based learning has been widely integrated in medical systems for its unprecedented performance. Various literature studies have been investigated for segmenting the COVID-19 patients including UNet++, FCN, U-Net and ResUNet. However, COVID-19 lesions segmentation is still considered as a critical challenge due to its diffused, patchy and scattered infectious distributions [28]. Furthermore, the generated contextual information does not properly converge into final reconstruction, thus, resulting in sub-optimal performances. Therefore, the goal of this paper is to propose an efficient and intelligent monitoring of COVID-19 patient’s using artificial network.

### B. Paper Contribution

In this paper, COVIDSys, an AI based system has been proposed that is capable of performing precise categorization of COVID-19 patients using three different algorithms. The intrinsic network of the proposed framework which includes Back Propagation (BP), Bayesian rule algorithm (BR) and viterbi schemes provides an immediate and effective solution by overcoming conventional approaches. The potential contribution of the paper is discussed as follows:

1) An integrated AI based mechanism is proposed combining the decision tree generation based on 15 symptomatic results of COVID-19 patient such as skin rash, fever, bleeding, score throat, eye pain, joint pain, muscle pain, nausea, fatique, itching, vomiting, abnormal pain, redness, breadth issue, cold of 2831 volunteers. The presented data set contains 180131 records containing source, target, start and end time of interaction with an interval of 24 hours.
2) The AI network is further combined with multi-layered stage having BP and BR schemes to generate an optimal and error free categorization of COVID-19 patient’s while analyzing their symptoms.
3) The viterbi algorithm is further integrated with BP and BR algorithms to select the best category of COVID-19 patients by improving the accuracy and susceptibility of the system [29].
4) The present study identifies the applicability and suitability of multi-layered network to further classify the patients into four different categories namely Infected (IN), Uninfected (UN), Exposed (EP) and Susceptible (ST).
5) The proposed system is simulated, analyzed and compared over various existing schemes including Random Tree (RT), Fuzzy C Means (FCM) and REPTree (RPT) methods against several performance measuring parameters.

The rest of this paper is organized as follows. A discussion on ANN techniques by different researchers is presented in Section 2. A secure ANN mechanism using BP, BR and Viterbi models is presented in Section 3. The validation of the proposed approach and its comparison is described in Section 4. Section 5 concludes the paper.

## II. Related Work

Chen *et al.* [30] have proposed a low-cost pervasive sensor by developing a novel signal algorithm after eliminating the un-necessary noise. The proposed model conducted the tests in different modes such as breathing, coughing and others for detecting the respiratory rates. The proposed mechanism achieved 98.98% respiration estimation and 97.33% of cough detection accuracy. The authors claimed the effectiveness of proposed system for screening the COVID-19 patient’s and enabling the large scale diagnosis and monitoring.

Rodriguez *et al.* [31] have used CNN and deep learning algorithms for determining the directions and path of a person by analyzing its threshold limit. The proposed approach results indicated the efficient implementation of the module by seeking its control of maximum population generated by SARS-COV-2 virus. The proposed approach tests were conducted on AMD Ryzen 7 3750H processor that determined the number of persons entering or exceeding the capacity permitted by pandemic on 50% of the original population.

Moura *et al.* [32] have proposed a novel automatic scheme for classifying the X-ray images into three different categories. The authors have applied three deep learning algorithms based on CNN architecture. The proposed scheme is validated against specific dataset by retrieving the various X-ray images of patient’s. The proposed system claimed 79.62%, 90.27% and 79.86% of global accuracy by facilitating the decision making progress in the system.

Laguarta *et al.* [33] have proposed an AI mechanism for identifying the COVID-19 patients through a voice recording model via smart phone. The authors have designed an AI speech recognition system by extracting the COVID-19 patient’s features. The authors have further provided a saliency map in real time scenarios for monitoring their conditions. The authors have used a learning algorithm to further test the proposed framework. The authors have validated the obtained results with the specificity and sensitivity of 94.2% and 98.5%. The authors have concluded to practically use case the worker, students, jobs and public transport sectors.

Hossain *et al.* [34] have proposed an B5G by utilizing the 5G networks having high bandwidth, low latency by detecting the CT scan and X-ray images of the persons. The authors have developed a surveillance mechanism to monitor the mask wearing, social distance and body temperature features. In addition, the authors have proposed three deep learning mechanism having deep tree, ResNet50 and inceptionv3 for investigating the patients. Further, they have used a blockchain network to ensure the security of each patient record in the hospital. Vedaei *et al.* [35] have presented a potential application of IoT along with social distancing from preventing the COVID-19 patient. The authors have proposed a three layer framework including IoT devices, Machine learning schemes and smart phone applications fir regularly monitoring the BP, blood oxygen, cough rate and temperature. The proposed frameworks guided the users for maintain the social distance and environmental hazards due to virus spread while updating the information regularly. The authors have considered two different scenarios for comparing the results in environmental constraints. The authors claimed to assistance the COVID-19 exposure.

Ndiaye *et al.* [36] presented the up to date survey on COVID-19 and illustrated how it affected the entire world. The authors have discussed the usage of latest technologies such as IoT and smart devices while tracking, tracing and spread mitigation of the virus. In addition, the authors have discussed the hardware deployment of systems to see the spreading pandemic. Further, the effects of COVID-19 on management and evolution of latest architectures have been discussed. The authors have provided the use of IoT techniques for surveillance and managing the COVID-19 patients. Further, the authors have also highlighted the future of IoT devices in global virus pandemic detection. Amin and Hossain [37] have illustrated a comprehensive literature on Edge and IoT smart healthcare system [38] by focusing on various published articles between 2014-2020. The authors have answered various concerns related to AI, IoT, cloud and edge computing and medical signals etc [39], [40]. In addition, the authors have addressed the ongoing challenges and future directions in this research.

## III. Proposed Trust Computation Mechanism

ANN can be defined as a machine designed to perform a task similar to the way brain functions. Recently, various ANN applications have been explored including in the field of health care management systems. The ANN has previously been used by many researchers in health care systems covering the data management, record security, interactions between doctor and patient and so on. In terms of analyzing the patient’s health, the ANN method to multi-objective optimization has offered efficient solution to further improve the decision making process. The ANN-based Computer Aided Designing (CAD) system is considered as a promising tool to generate immediate and accurate results for patients’ health by assisting the biological neurons. Though ANN-CAD systems have been studied over last two decades, however, most of the literature still highlights only the various common concerns such as:

• Few previous studies focus on using ANN for managing or recording the patients’ data in healthcare systems. The management systems are determined by analyzing the obtained models.
• Previous healthcare systems focus on categorizing the patients into two different categories i.e. healthy and infectious.

Building from the earlier described work, the paper further attempts to improve the classification quality for COVID-19 patients’ healthcare categorization. The proposed system has the mentioned categories:

• The COVID 19 patient classification is carried based upon 15 different symptoms suffering. The mentioned 15 symptoms have been proven [41] as input to classify the patient’s category.
• This paper proposes the use of 15 symptoms of COVID-19 as input for ANN to categorize the patient’s health automatically.
• The present study identifies the applicability and suitability of multi-layered network to further classify the patients into four different categories namely Infected (IN), Uninfected (UN), Exposed (EP) and Susceptible (ST).

### A. Multi-Layered Perceptron (MLP) Network

ANN is a computational system that is inspired by the biological concept of neural networks called neurons. The neurons are miniature cells that our brain is made of. A biological neuron is determined as an assortment of billions of neurons that are considered as the base for ANN to model biological structures in terms of both operation and architecture. ANN works as a mathematical computational model for data classification, non-linear function and non-parametric/clustering regression. The ANN is capable in providing performance reliability in automatic decision making process. Multi-layered perceptron is considered as one of the most commonly used ANN type.

**FIGURE 1.**
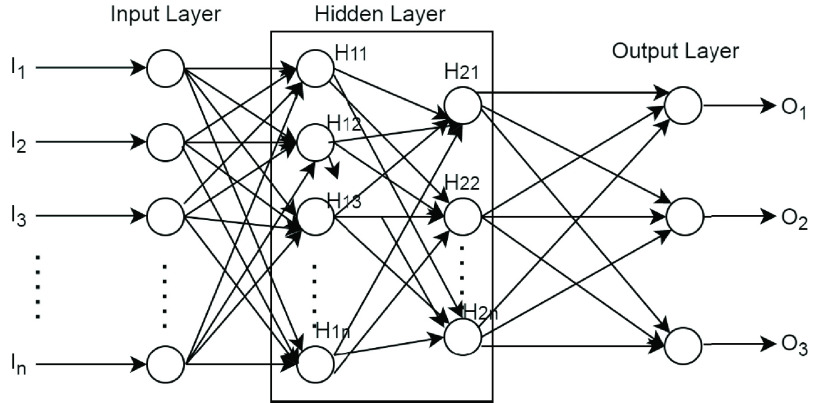
Multi-layered perceptron architecture network.

Figure 1 depicts the perceptron model proposed by Rosenblatt in 1958 in a layer to form a network. The neural network depicted in Figure 1 is known as MLP architecture consisting of n outputs, $m_{h}$ hidden and $m_{i}$ input modes that can be expressed as equation 1:

\begin{equation*} Y_{z}(t)=\sum _{y=1}^{m_{h}} W_{yz}^{2} F (.) \sum _{x=1}{m_{i}}W_{xy}^{1} P_{x}(t)^{0} + b_{y}^{1}\tag{1}\end{equation*}

$1 < =z < =n$ where $w_{xy}$ and $w_{yz}$ denote the weight connection between input, hidden layer and output layer respectively. $F(.)$ is defined as an activation function that is normally selected as a sigmoid function. In addition, $b_{y}^{1}$ represents the hidden value threshold that is supplied to input layer.

From presented in equation 1, $w_{xy}$, $w_{yz}$ and by values are determined using an appropriate algorithm consisting of back propagation (BP), viterbi or Bayesian rule (BR) algorithms. The BP algorithm is commonly used to determine the optimum value because of its efficient implementation and performance. The BP algorithm is based on error-correction rule that automatically analyzes, categorizes and corrects the patients’ category. In addition, two more algorithms called Bayesian and viterbi algorithms are used to train the MLP architecture network.

### B. Back Propagation Algorithm

BPA is the commonly used gradient descent method that generates the derivation values by modifying the weights according to learning rate parameters. The BPA is the steepest descent method where weights among $y^{t}h$ neuron hidden layer and $x^{t}h$ neuron input layer are respectively modified according to:

\begin{align*} w_{xy}(t)=w_{xy}(t-1) + \delta w_{xy}(t) \\ b_{y}(t) = b_{y} (t-1) + \delta b_{y}(t)\end{align*}

The increment $\delta w_{xy}(t)$ and $\delta b_{y}(t)$ are given by:

\begin{align*} \delta w_{xy}(t)= \eta _{w} P_{y}(t) P_{x}(t) + \alpha _{w} \delta w_{xy}(t-1) \\ \delta b_{y}(t)= \eta _{b} P_{y}(t) + \alpha _{b} \delta b_{y}(t-1)\end{align*}

where, $b$ and $w$ subscripts denote the threshold and weight respectively. While $\alpha _{b}$ and $\alpha _{w}$ are considered as the momentum constants that illustrate the persuade of changes in previous parameters on the movement of direction in parameter space.

In addition, $\eta _{b}$ and $\eta _{w}$ determine the learning rates and $P_{y}(t)$ determines the error signal of $y^{t}h$ hidden layer neurons that is propagated in the network. Since, the activation function of the output layer is linear, the error signal at output node is defined as in equation 2:

\begin{equation*} P(t)= y_{z}(t)-y_{z}'(t)\tag{2}\end{equation*}

where $y_{z}(t)$ is the expected putput. Further, the hidden layer neurons are defined by equation 3:

\begin{equation*} P_{y}(t)= F'(P_{x}(t)) w_{yz}^{2}(t-1)\tag{3}\end{equation*}

where $F'(p_{x}(t)$ is the first derivation of $F(p_{x}(t)$ with respect to $p_{x}(t)$. Since the BP is steepest descent method, the BP algorithm endures from a slow convergence rate. The BP algorithm can be sensitive at selected parameters and global minima may be trapped at local minima.

### C. Viterbi Algorithm

Due to various behaviours of COVID-19 patients, the graph of each patient will be different according to their updated activity of recovery. The viterbi algorithm is used to compute the probability of all states in hidden layer based upon their emissions and activities in the environment. Algorithm 1 illustrates the viterbi algorithm steps for categorizing the patients based upon their recovery rate. It uses the maximization function at each time instance to select/elect the best category of patient according to the recovery rate. Let $\sigma $ ensures the maximum probability of a patient to end in a state $i$ with request sequence of length ‘$l'$ that results in first ‘$o'$ observations of hidden layers then $\sigma _{t}(i)$ can be defined as in equation 4:

\begin{align*}&\sigma _{t}(i)= max Pr(q_{(1) }, q_{(2) }\ldots. \\&\qquad \qquad \qquad \qquad \,\,q_{(t-1}; o(1), o(2) \ldots..o(t)|q(t)=r_{i})\tag{4}\end{align*}

Algorithm 1 **Viterbi Algorithm**
Begin
Step 1: Initialization of matrix and probability variable as:
  \begin{align*} \sigma _{1}(i)=Pr_{i}b_{i}(o(1)) \\ \delta _{1}(i)=0\end{align*}
Step 2: Recurring for computing the requested arguments of the patient’s by updating the output in $\delta $.
  \begin{align*} \sigma _{t}(j)=max_{i}[\sigma _{t-1}(i)\alpha _{ij}]b_{j}(o(t)) \\ \delta _{t}(j)=max_{arg_{i}}[\sigma _{t-1}(i)\alpha _{ij}]\end{align*}
Step 3: The recursion step termination based on given conditions:
  \begin{align*} p^{*}=max_{i}[\alpha _{T}(i)]\\ q_{T}^{*}=max_{arg_{i}}[\alpha _{T}(i)]\end{align*}
Step 4: The best state is opted by backtracking as:
  \begin{equation*} q_{T}^{*}=\delta _{t+1}(q_{t+1}^{*}\end{equation*}
End

Table 1 represents the notations of viterbi algorithm implementations:

**TABLE 1:** Viterbi Algorithm Notations

Symbol	Meaning
$\sigma _{i}(t)$	Prob. of patient to end in state ‘i’ based upon its request length ‘l’
$b_{i}(o(t))$	Prob. of output o(t) upon initial state ‘i’
$a_{ij}$	Prob. transition from state i to state j.

The Viterbi algorithm is detailed as:

### D. Bayesian Rule Algorithm

The Bayesian rule algorithm is given as equation 5:

\begin{equation*} P(\phi |I)=\frac {P(I|\phi)}{P(I)}\tag{5}\end{equation*}

where, $P(\phi)$ is the prior probability of parameter $\phi $ and $P(\phi |I)$ is the probability of information $I$.

The Baye’s rule was generally used to illustrate the posterior probability of o given information $I$. This offers an entire distribution over possible o values. The given process is applied over ANN by defining the probability distribution over weights w and training information $P(w|I)$. The posterior distribution over weights is further determined by equation 6:

\begin{align*} P(w|I)=&\frac {P(I|w) P(w)}{P(I)} \\ P(w|I)=&\frac {P(I|w)}{P(I|w) P(w) dw}\tag{6}\end{align*}

**FIGURE 2.**
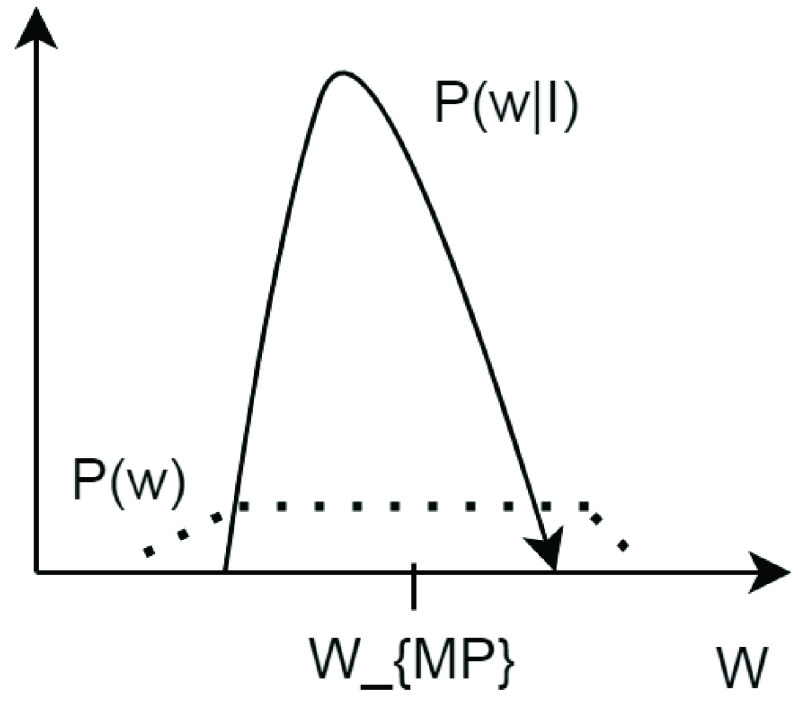
Changing prior weights to posterior weights.

In Bayesian rule algorithm, the weights learning determines the belief changing about prior weights $P(w)$ to posterior $P(w|I)$ as a consequence of information seeing as depicted in Figure 2. The mentioned Figure 2 illustrates the change of weights by determining or analyzing the patient’s category according to BR algorithm. Further, the analysis of weights comparison between prior and posterior values are determining by varying the time and the probability rate.

**FIGURE 3.**
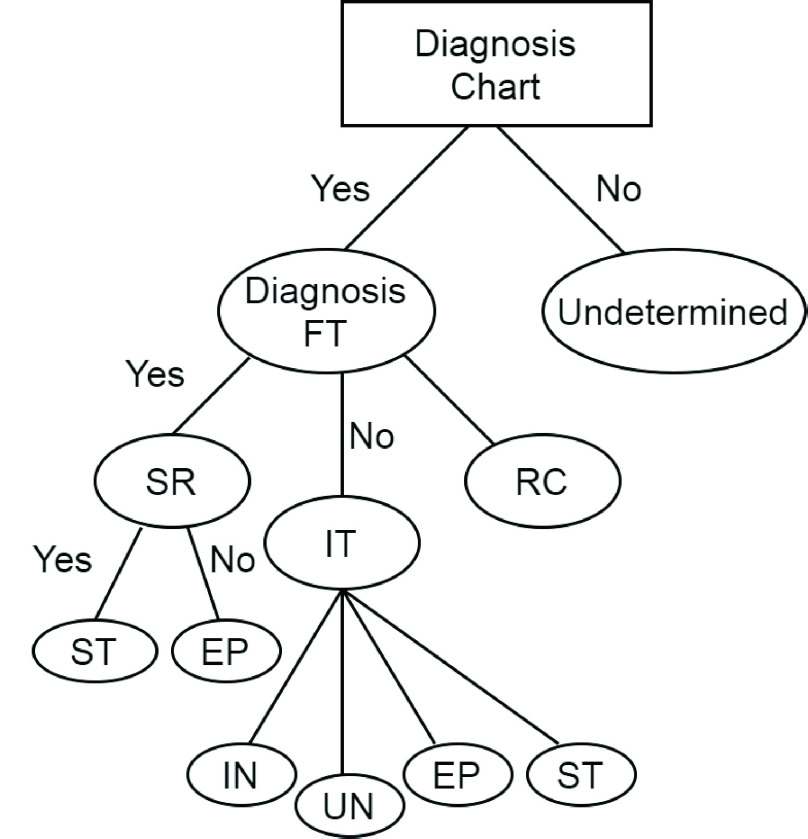
Tree visualization of COVID-19.

**FIGURE 4.**
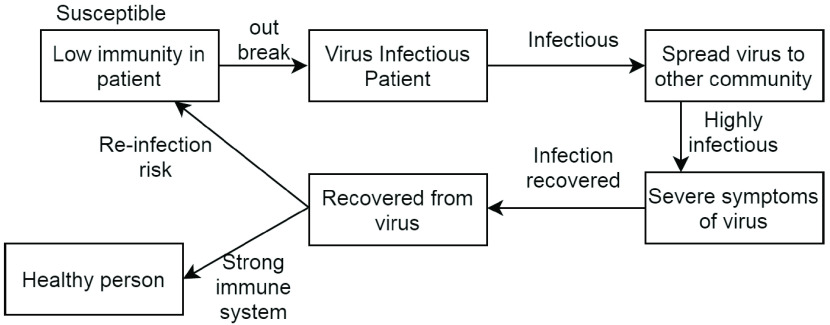
COVID-19 patient life cycle outbreak.

Figure 3 and Figure 4 represent the tree visualization and life cycle of COVID-19 patient in Weka. Figure 3 represents the decision tree after classifying the data components categorizes the patients into IN, UI, EP and ST categories. As represented in Figure 3, the user is separated as susceptible if the patient shows symptoms such as cold, fever, throat pain etc.

In addition, the category EP represents that the user is infectious at the present stage by recognizing the initial symptoms. Further, IN is the one where the patient shows the complete symptoms of the virus and has maximum infection that can be contagious to other people. Furthermore, UN means that the person does not have any symptom and is clear from the infection.

Further, Figure 4 depicts the life cycle of COVID-19 patients where the patient’s can be categorized into healthy and infectious category by analyzing their infection risks and immune system response. The depicted Figure 4 represents the analysis of the person’s category by leading it into an susceptible state by following certain predefined procedures.

## IV. Performance Evaluation

### A. Experimental Results

This paper study applies similar analysis for classifying the data done by [27], [42]. Initially, the optimum analysis is undertaken and experimented to illustrate the hidden number of nodes that may generate best COVID-19 patient classification. For the numerical analysis, the multi-layered network is trained over 3600 epochs with the time taken of 9.763 s, 5.248 s and 4.321 s using BP, BR and viterbi algorithms. Table 2 depicts the results and obtained various training methods.

**TABLE 2:** Optimized Structure Results Using BP, BR and Viterbi Algorithms

Multi-layered Algo	Optimized Hidden Nodes
BP	8
BR	4
Viterbi	4

The experimental analysis is categorized over three different sections namely, data integration/acquisitions, decision tree and graph-based evaluation as detailed in [27], [42].

• Data acquisition and integration: Symptom-based data set is attained including 15 attributes such as skin rash, fever, bleeding, score throat, eye pain, joint pain, muscle pain, nausea, fatique, itching, vomiting, abnormal pain, redness, breadth issue, cold of 2831 volunteers. The 5124 data set cases having environment attributes are obtained from including daily reports generated from [42]. The symptom generated data set is combined with environment to test the proposed system. The presented data set contains 180131 records containing source, target, start and end time of interaction with an interval of 24 hours.
• Decision tree: The decision tree is used to categorize the patients into IN, UN, ST and EP that is further implemented on weka. Various statistical parameters are considered to examine the generated records of COVID-19 patients.
• Specificity: It is called as false positive rate and it categorizes the percentage of patients who are diagnosed incorrectly by the system.
• Sensitivity: It is defined as true positive rate and considers the patients who are correctly identified by the system.
• F-measure and ROC: These are used to determine the accuracy of classifying the proposed scenario.

In addition, the performance comparison is done by doing the second analysis phase depending upon the first analysis. The analysis is done through an accurate classification of COVID-19 patients for testing and training the data. The classified details of decision tree and the results of the proposed framework under different training schemes are represented in Table 3 and 4.

**TABLE 3:** Classification Accuracy of Decision Tree

Class	IN	UN	ST	EP
Specificity	0.00	0.041	0.058	0.021
Sensitivity	1.00	0.891	0.876	0.832
Precision	1.00	0.732	0.612	0.613
Recall	1.00	0.871	0.513	0.589
F-Measure	1.00	0.701	0.683	0.732
ROC	1.00	0.673	0.611	0.512

**TABLE 4:** Classification Accuracy of Decision Tree

Multi-Layered network	Training	Testing	Overall	Epochs No.	Time (sec)
BP	68.87	71.31	61.20	35000	9.895
BR	93.51	84.78	91.50	10	4.362
Viterbi	89.32	88.23	90.89	10	3.512

Table 3 represents the evaluated values of various classified patient’s that are categorized according to various classes such as specificity, sensitivity, precision, recall, F-measure and ROC. The depicted Table 3 represents the evaluated value of proposed mechanism using BP, BP and Viterbi algorithms. Further, Table 4 represents the individual training and testing data values of a; the proposed algorithm over number of epochs and time required to run that particular algorithm to categorize or classify the patient’s category into various types.

### B. Results and Discussion

**FIGURE 5.**
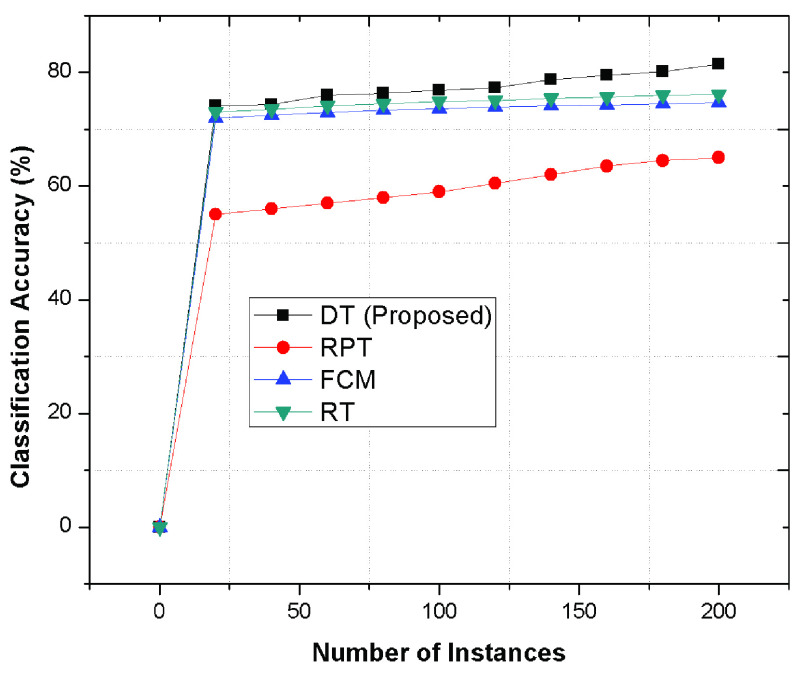
Classification Accuracy to determine the patient’s category.

Initially, the various classification algorithms are analyzed including two statistical measures, namely, classification time and accuracy. The classification accuracy is depicted in Figure 5 that determines that the decision tree using BP, BR and Viterbi algorithms performs better as compared to Random Tree (RT), Fuzzy C Means (FCM) and REPTree (RPT) methods. The classification of the data according to their behaviours are efficiently determined using BP and BR algorithms to select best category of COVID-19 patient’s by improving the susceptibility and accuracy of the system. The out performance of proposed mechanism is due to BP algorithm that ensure the accuracy and specificity in the network while classifying the patient’s into certain categories.

**FIGURE 6.**
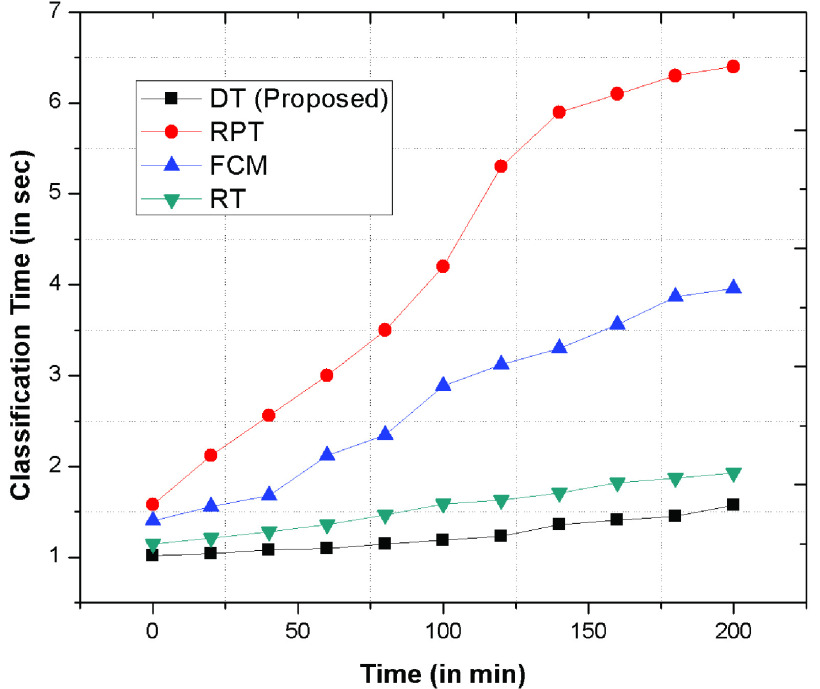
Classification time required to category the COVID-19 patient’s.

In addition, Figure 6 depicts the classification time of decision tree, RPT, FCM and RT using various data-sets. The time required to categorize the patient’s into IN, UN, EP and SP is less as compare to other existing schemes because of BP, and BR schemes which generates an optimal and immediate category by recognizing their symptoms. The depicted graph concludes that the decision tree takes less classification time as compared to existing schemes. The reason is due to accurate classification using probabilistic scenarios using Viterbi algorithm that may further provides an error free and immediate categorization of patient’s.

**FIGURE 7.**
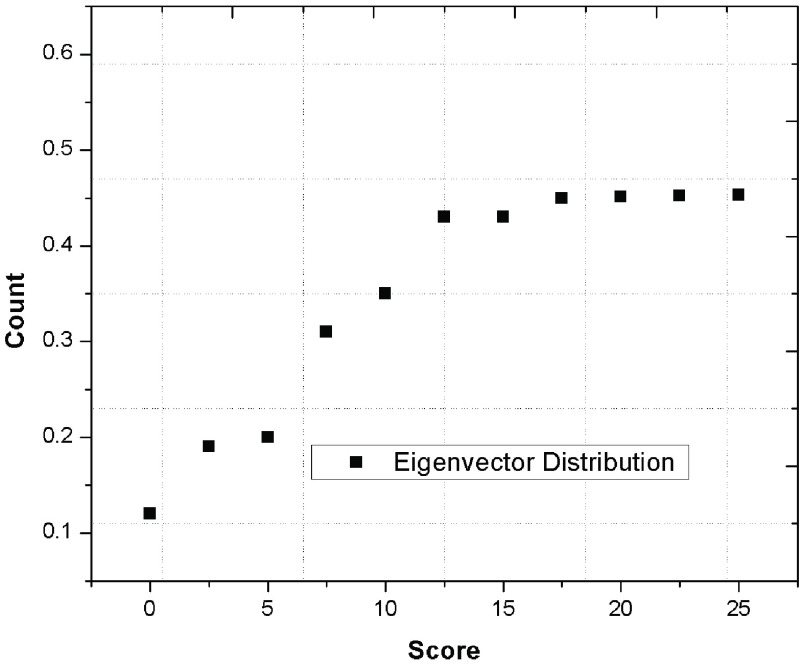
Eigen vector distribution to analyze the smooth distribution of the data.

Further, Figure 7 represents the eigen vector distribution of the proposed graph which explains how the data is smoothly distributed in the network and further categorized widely by the proposed phenomenon. The integration of multi-layered schemes along with Viterbi algorithm improves the data distribution for reducing the classification time of the patient;s category.

**FIGURE 8.**
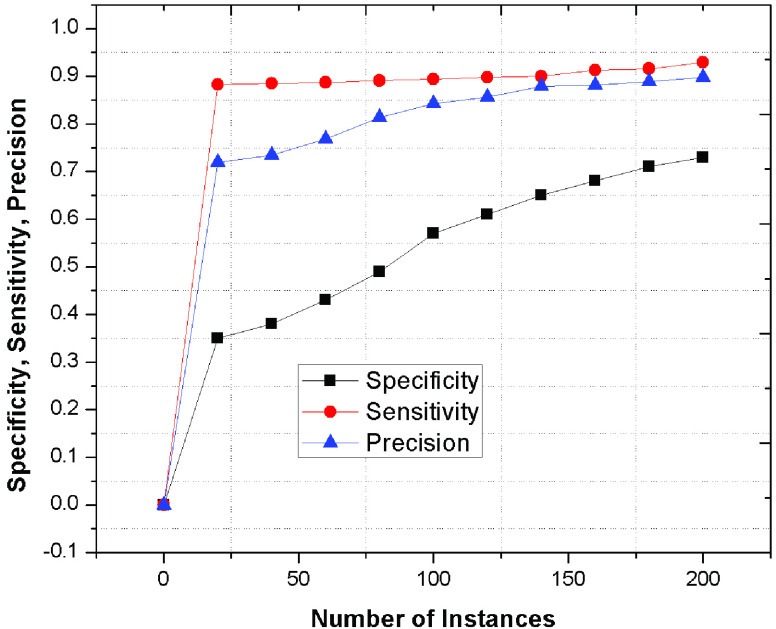
Specificity, Sensitivity, Precision values of proposed phenomenon.

Furthermore, Figure 8 represents the specificity, sensitivity and precision of the proposed framework as compared to the existing schemes. he proposed mechanism outperforms because of its accurate judgement of patients’ classification by identifying various symptoms through artificial neural network. However, the existing schemes provides less accuracy with significant delay while analyzing the symptoms to categorize the patient’s as compared to proposed approach. In addition, the integration of Viterbi algorithm with BP and BR ensures an accurate and efficient classification mechanism.

**FIGURE 9.**
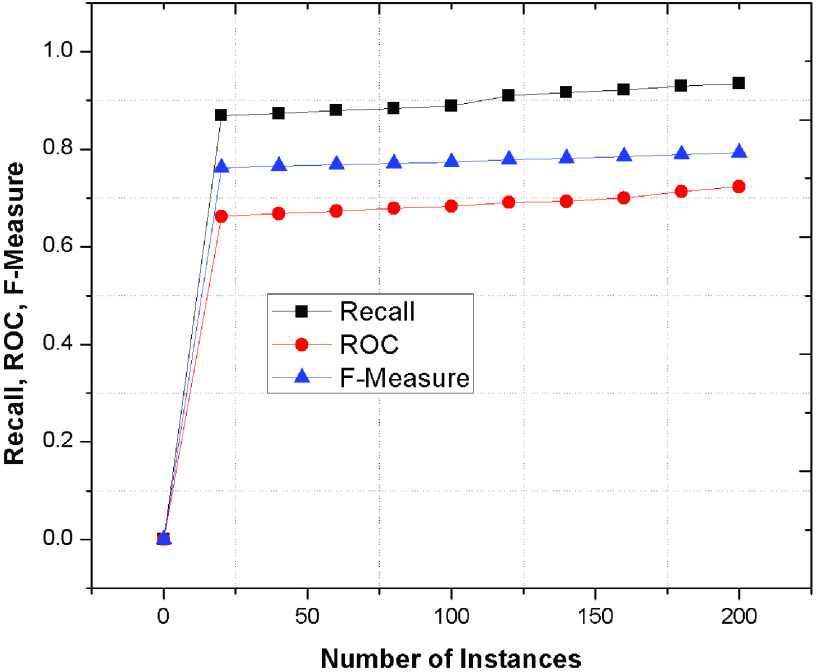
Recall, ROC, F-Measure value of proposed phenomenon.

Finally, Figure 9 depicts the recall, ROC and F-measure parameters of the proposed scheme as compared to the conventional FCM, RT and RPT methods. The following parameters determines the accuracy of categorizing the patient’s through ANN as compare to existing schemes because of integrated system of BP, BR and Viterbi algorithm. The depicted values represents significant improvements as compare to conventional mechanisms because of accurate decision and recognition of patient’s symptoms using error free classification using BP algorithm.

## V. Conclusion

This paper aims to analyze the categories of COVID-19 patients with respect to various time events. The paper determines the ANN network in terms of health monitoring and result analysis with the biological scrutinizing approach. The paper has used BP, BR and viterbi algorithms to illustrate the progress and monitoring of COVID-19 patients’ category on timely basis. The proposed mechanism validates the suitability of AI systems and is capable of classifying the COVID-19 patients based on various symptoms. The experimental results of the proposed mechanism depict higher accuracy and less response time for various patient categories using decision tree.

In addition, further analysis and improvements can be made to upgrade the proposed scheme. Various case studies may be done to test the reliability and capability of the system. Another good implication is by using various AI networks and engaging learning mechanisms.
